# Light Weighted Healthcare CNN Model to Detect Prostate Cancer on Multiparametric MRI

**DOI:** 10.1155/2022/5497120

**Published:** 2022-05-28

**Authors:** Mukesh Soni, Ihtiram Raza Khan, K. Suresh Babu, Syed Nasrullah, Abhishek Madduri, Saima Ahmed Rahin

**Affiliations:** ^1^Senior IEEE Member, Bhopal, India; ^2^Academician, Jamia Hamdard Delhi, Delhi, India; ^3^Department of Biochemistry, Symbiosis Medical College for Women, Symbiosis International (Deemed University), Pune, India; ^4^Department of Information Systems, College of Computer Engineering & Sciences, Prince Sattam Bin Abdulaziz University, Al-Kharj 11942, Saudi Arabia; ^5^Engineering Management, Duke University, NC, Durham, USA; ^6^CSE College, United International University, Dhaka, Bangladesh

## Abstract

The SEMRCNN model is proposed for autonomously extracting prostate cancer locations from regions of multiparametric magnetic resonance imaging (MP-MRI). Feature maps are explored in order to provide fine segmentation based on the candidate regions. Two parallel convolutional networks retrieve these maps of apparent diffusion coefficient (ADC) and T2W images, which are then integrated to use the complimentary information in MP-MRI. By utilizing extrusion and excitation blocks, it is feasible to automatically increase the number of relevant features in the fusion feature map. The aim of this study is to study the current scenario of the SE Mask-RCNN and deep convolutional network segmentation model that can automatically identify prostate cancer in the MP-MRI prostatic region. Experiments are conducted using 140 instances. SEMRCNN segmentation of prostate cancer lesions has a Dice coefficient of 0.654, a sensitivity of 0.695, a specificity of 0.970, and a positive predictive value of 0.685. SEMRCNN outperforms other models like as V net, Resnet50-U-net, Mask-RCNN, and U network model for prostate cancer MP-MRI segmentation. This approach accomplishes fine segmentation of lesions by recognizing and finding potential locations of prostate cancer lesions, eliminating interference from surrounding areas, and improving the learning of the lesions' features.

## 1. Introduction

Prostate cancer (PCa) is one of the most frequent kinds of cancer in middle-aged and older men. According to the most current epidemiological statistics [1–6], the incidence of PCa has grown dramatically in my nation during the last several years. Magnetic resonance imaging (MRI) (multiparametric magnetic resonance imaging, MP-MRI) is a common approach for the early detection of prostate cancer [6–8].

Accurate segmentation of the area is critical for determining the lesion's malignancy and directing the biopsy. Manual delineation of the prostate cancer lesion region, on the other hand, requires specialist skill and is extremely time-consuming [9]. Segmentation of the prostate cancer lesion region is detected automatically and precisely by a computer. It is vital, but currently confronts the following three obstacles: (1) significant variation in the shape and size of prostate cancer lesions across individuals; (2) the borders of prostate cancer lesions are muddled; and (3) numerous prostate cancer lesions, that is, a patient's prostate may have multiple prostate cancer foci.

Recent years have seen a surge in the usage of image segmentation algorithms based on deep learning for automated lesion segmentation on medical pictures, with promising results [10–12]. Segmentation features may be classified into techniques for image segmentation based on pixel classification and algorithms for image segmentation based on area classification [13]. The literature [9] presented a deep learning network composed of an adversarial neural network and a U net [14] for the automated segmentation of prostate cancer lesions using MP-MRI. The proposed method plays a crucial role for early detection of prostate cancer sites. The prostate region only takes up a minor portion of an MRI picture, which contains several human tissue components. Other tissue structural information can readily interfere with directly segmenting prostate cancer lesions on prostate MRI images. Semantically segmenting the lesion region yields a Dice coefficient of 0.410.28 and a sensitivity of 0.550.36. While this approach is good in detecting the approximate location of prostate cancer lesions, segmentation accuracy could be improved.

Unlike the pixel-based image segmentation approach, Mask-RCNN uses the region-based image segmentation method [15]. As a result, when Mask-RCNN (MRCNN) is used for prostate cancer lesion segmentation, it may identify suitable regions with lesions and reduce the requirement for fine segmentation. As a consequence, the spectrum of prostate cancer lesions is optimized to increase the accuracy of segmentation.

While MRCNN is capable of extracting convincing aspects of the prostate cancer lesion region, integrating the information with MP-MRI efficiently requires more investigation. The MRCNN is a method for segmenting prostate cancer lesions that can find acceptable locations with lesions and lessen the need for precise segmentation. As a result, the spectrum of prostate cancer lesions has been modified to improve segmentation accuracy. The proposed network is utilized as the foundation for detecting and analyzing potential areas harboring prostate cancer lesions. To maximize the use of the current network model, MP-MRI was converted to RGB pictures. Of three channels are employed to combine MP-MRI data at the level of the input picture. The literature [16] extracted the apparent diffusion coefficient (ADC) and T2-weighted (T2-weighted) using two parallel convolutional networks. (T2W) image features, guiding the convolutional network to extract useful features from distinct MRI sequences during network training by leveraging the difference between ADC and T2W feature maps as constraints. Multiple parallel volumes were employed in the literature [17]. The product network removes distinct lines of MRI features, and feature concatenation is utilized to fuse the MP-MRI information. Although these approaches can train the network model concurrently with MP-MRI, properly integrating the high-level features into MP-MRI is difficult, and so the model accuracy has to be improved.

This article presents a method for picture segmentation based on SE region categorization by automatic segmentation of prostate cancer lesions on the MP-MRI prostate area using Mask-RCNN. The MRCNN network is utilized as the foundation for detecting and analyzing potential areas harboring prostate cancer lesions. It is suggested to extract ADC and T2W image features using two parallel convolutional networks, respectively, and to analyses the difference between the ADC and T2W image features using a squeeze-and-excitation block (SE-block). For identifying the lesion's malignancy and directing the biopsy, accurate segmentation of the region is crucial. Manual delineation of the prostate cancer lesion location, on the other hand, necessitates a high level of expertise and takes a long time. Correlation modeling; leverage complementarily in information from distinct sequence MRIs; automatically calibrate the weights of varied feature channels via explicit learning; enhance valuable features and suppress invalid features; and successfully merge practical knowledge in MP-MRI. The experimental findings demonstrate that the suggested SEMRCNN may significantly increase the accuracy of prostate segmentation for cancer lesions. If we compare the proposed model with existing model, we find that the proposed method is more capable for identifying prostate cancer sites from multiparametric magnetic resonance imaging areas automatically (MP-MRI). And the use of extracted features to offer fine segmentation based on candidate regions is investigated.

The present article has been planned into various sections. Section 1 deals with introducing the concept and importance of multiparametric magnetic resonance imaging (MP-MRI).  Section 2 puts light on automatic segmentation network of prostate cancer lesions based on SE-Mask-RCNN. Section-3 illustrates the experimental analysis of the proposed research. The result band analysis is described in Section 4. Finally, Section 5 portrays the conclusion and possible future works based on the proposed framework.

## 2. Automatic Segmentation Network of Prostate Cancer Lesions Based on SE-Mask-RCNN

### 2.1. MRCNN Deep Learning Network

**Figure 1:**
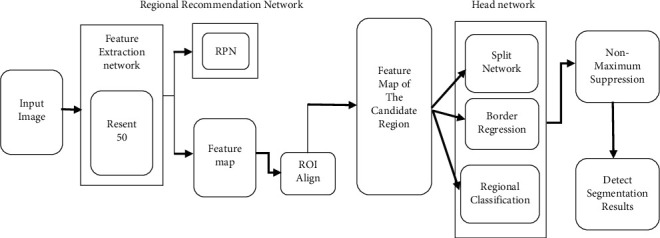
MRCNN network structure.

Deep learning networks, as seen in Figure 1, combine target detection with precise segmentation and are made up of the following order: (1) Feature extraction network; (2) RPN (region proposal network); and (3) HN (head node). Use the Resnet50 feature extraction network to extract numerous feature maps with varying levels of detail from the picture. RPN creates potential detection targets in the form of candidate areas. The MRCNN finds and selects candidate areas containing detection objectives, then refines the segmentation of target objects in those areas. It also serves as a basis for recognizing and evaluating probable prostate cancer tumors. The RoI Align layer generates the feature map of the candidate region. Using a map, the candidate region's key features revisions are made in the head network by a border regression branch network the category and potential of a target in that region are provided by a classification branch; and finally, a segmentation network provides a segmentation result for that target. Use nonmaximum suppression to eliminate candidate regions with a low probability of object existence or high intersection and get final candidate regions and segmentation results.

Compared with image segmentation networks based on pixel classification, MRCNN locates and extracts candidate regions containing detection targets and performs more refined segmentation of target objects in the candidate regions.

### 2.2. SE-Block

SE-block [18] suggested by literature [18] simulates the dependency of distinct feature channels during the feature extraction process and recalibrates the weights of altering features. Global pooling is used to compress the spatial information of different feature channels into a channel descriptor; two fully connected layers and one ReLU layer model the interdependence between other feature channels and obtain the weights of different feature channels through sigmoid activation function. It is important to remember that by scaling, you are increasing the relevance of the feature channel while simultaneously suppressing the irrelevant feature channel, giving your deep learning network better access to learning valuable features on-the-fly.

### 2.3. SEMRCNN Deep Learning Network

**Figure 2:**
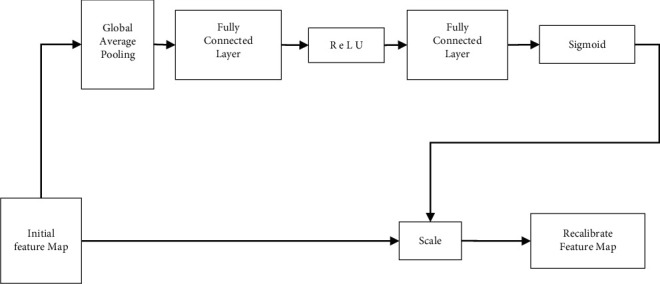
SE-block structure diagrams.

To take advantage of the practical information contained in mp-MRI and to improve the segmentation accuracy of prostate cancer lesions, a deep convolutional neural network SEMRCNN is proposed. This network is based on region classification and feature extraction via a two-channel convolutional network.  Figure 2 shows SE-block structure diagrams.

The overall framework of SEMRCNN and MRCNN is the same, mainly composed of three parts: feature extraction network, RPN network, and head network. The feature extraction network extracts the input image feature map, and the RPN Candidate regions give the features on the graph. The candidate region feature map is obtained through the RoI Align layer and input to the head network. The head network evaluates the probability of lesions in the candidate region, corrects the position and size of the candidate region, and segments the prostate cancer lesion region in the candidate region. The maximum value suppression method removes the candidate regions with a small probability of lesions and candidates with a high degree of overlap. It obtains the final candidate region and lesion segmentation results. The convolution neural network is frequently employed in healthcare for a number of purposes, particularly in cardiologist. Diagnoses, electronic signal interpretation, clinical imaging evaluation, and radiography have all benefited from the use of CNN. The location and direction of an image are not encoded by convolutional neural network. It is necessary to collect a large amount of training data.

**Figure 3:**
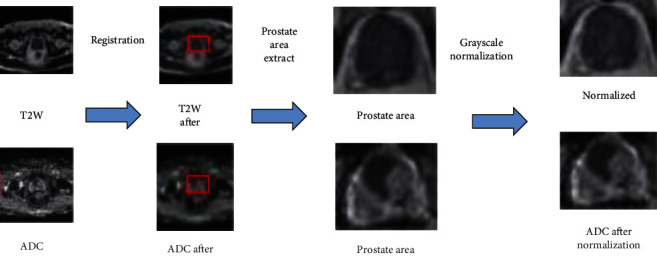
Data preprocessing example.

**Figure 4:**
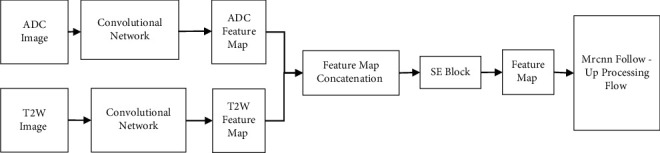
SE-block improved MP-MRI information fusion methods.

Different from the MRCNN network, SEMRCNN adopts the proposed SE-block improved MP-MRI information fusion method to construct a new feature extraction network, as shown in Figure 3. SE-Resnet is used as in Figure 4. The convolution network is implemented by the proposed MP-MRI information fusion method.

As shown in Figure 3, the SEMRCNN feature extraction network uses two parallel SE-Resnet networks to extract three-level feature maps of different sequences of MRI images, respectively, and form concatenated feature maps in an attached manner. Using SE, the block models the correlation between the other feature channels of each tandem feature map, automatically calibrates the weights of different feature channels, improves the valuable features in the tandem feature map and suppresses irrelevant features, and obtains a fusion feature map that incorporates the valuable information of MP-MRI.

SE-convolution Resnet's layer analyses the input picture using a 77 convolution kernel with stride 1, while the max-pooling layer employs a 33 pooling operation. Each residual block is built of three convolutional layers and has a similar structure. Convolution layer 1 and convolution layer 3 convolution operations use a convolution kernel of 11, whereas convolution layer 2 convolution operations use a convolution kernel of 33. The stride of convolution layer 2 in residual blocks 3 and 5 is adjusted to 2 to enable feature map downsampling. Both convolution layer 1 and layer 3 have a stride of one. The step size of all convolution layers is set to one in the difference block. In terms of performance and computational efficiency, just one SE-block is added after the residual blocks 2, 4, and 6, and three effective features on feature maps at various levels. The limited feature map is integrated with the high-level feature map to generate a balanced multiscale feature map that accounts for both image detail and generic information. The extracting features network obtains the feature map from the input picture, and the RPN Candidate regions provide the features on the graphs. The high-level feature map is upsampled and combined with the low-level feature map to create a balanced multiscale feature map that takes both picture detail and general information into account.

## 3. Experimental Analyses

### 3.1. Data Selection

ADC and T2W images of 140 prostate cancer patients from the Second Affiliated Hospital of Soochow University were retrospectively analyzed between January 2015 and March 2017. Among the 140 patients, the mean age of the patients was 74.4 years, and the range was 51–93. Of these patients, 129 had 1 prostate cancer lesion in the prostate region, 11 of the patients had 2 prostate cancer lesions for a total of 151 lesions. For each prostate cancer patient MP-MRI data, the radiologist selected 1–3 ADC and T2W image pairs for clearly visible prostate cancer lesions.

### 3.2. Data Preprocessing

A total of 273 ADC-T2W image pairs as a dataset selected from January 2015 to 2016219 ADC-T2W image pairs of 112 patients between October were used as a training set, and 27 ADC-T2W images of 14 patients were randomly selected from the remaining 28 patients between November 2016 and March 2017 27 ADC-T2W image pairs from 14 patients are left as the test set. Figure 4 shows an example of the process of registration, prostate region extraction, and grayscale normalization in data preprocessing.

To effectively utilize the information on ADC and T2W images, it is necessary to perform registration operations on ADC and T2W images. Literature [19] proposed a method using coordinate information stored in DICOM images for registration in the case of small prostate deformation. This method has the advantages of simplicity and effectiveness. The patient data used in this study are all screened by deformation; the conditions for registration using interactive coordinate information are met. Registration is performed using coordinate data stored in different sequences of MRIs.

Prostate MRI images contain many human tissue structures, and the prostate area only occupies a small part of the image. Segmenting prostate cancer lesions directly on prostate MRI images is easily interfered with by other tissue structure information, resulting in poor segmentation results. Manual selects the prostate region, and the size of the box should cover the entire prostate region to minimize the background influence.

Since there are certain differences in the gray distribution range of prostate mp-MRI between different patients, to reduce the influence of the difference in the gray distribution range on the automatic segmentation results of prostate cancer lesions, the gray levels of the mp-MRI images in the prostate region were analyzed separately standardized [20].

In the training set, the data is augmented using the rigid augmentation method of left-right and up-down flipping and the nonrigid augmentation method of sliding least squares transformation. Ten points are randomly selected for each training image as the initial point set. Each point in the point set.

The abscissa is randomly shifted by *x* pixels, and the ordinate is shifted by *y* pixels (the value of *x*, *y* is [−5, 5]) to generate the transformed point set. Calculate the related correspondence between the initial point set and the transformed point set. The moving most minor squares change [21], which is used on the original image to generate nonrigid augmented data. Augmented by nonrigid data, increase the amount of data in training set to 5 times the original. During the training process, use online data augmentation to perform rigid expansion of each batch's left and right and upside-downs. This study does not include the validation and test sets; use data augmentation.

### 3.3. Model Training

Utilize the Adam optimizer to apply the gradient descent technique throughout the training phase to determine the network parameters that minimize the error function. Set the initial learning rate of the network to 0.000 1 and the epoch to 100. The duration for network training is 50; and the learning rate is lowered to 0.000 05. The batch size is configured to be two.

On the same Windows 10 PC, all models are trained, verified, and tested. The PC is equipped with an Intel i7-9700 k processor running at 3.60 GHz and an NVIDIA GTX 1080Ti graphics card including 11 GB of video RAM. *Python* 3.6, CUDA 10.1, Keras2.2.4, and tensorflow1.10.0 are used as the core software environments.

### 3.4. Evaluation Indicators

For each prostate cancer patient data case, two experienced doctors manually delineated the commonly recognized prostate cancer lesion area on mp-MRI, and the delineation result was taken as the common area of the prostate cancer lesion. For the segmentation task, the Dice similarity coefficient (DSC) is one of the most widely used evaluation indicators. The calculation formula is

(1) $$\begin{array}{c} \text{DSC}=\frac{2\left|P\cap G\right|}{\left|P\right|+|G|}. \end{array}$$

In the formula, P represents the predicted lesion area and *G* represents the doctor's outlined lesion area.

Sensitivity may efficiently depict the segmentation findings' sensitivity to the lesion region. The specificity may be used to accurately determine the false-positive rate. Finally, the positive predictive value (PPV) may be used to determine the fraction of observed positive lesions that are genuinely lesions. As a result, the following formulae for sensitivity, specificity, and positive predictive value are used:

(2) $$\begin{array}{c} \text{sensitivity}=\frac{\text{TP}}{\text{TP}+{\text{FN}}^{\prime }},\\ \text{specificity}=\frac{\text{TN}}{\text{TN}+{\text{FP}}^{\prime }},\\ \text{PPV}=\frac{\text{TP}}{\text{TP}+{\text{FP}}^{.}}. \end{array}$$

The number of pixels correctly categorized as lesions is referred to as true positive (TP), the number of pixels correctly classified as normal tissue is referred to as true negative (TN), and the number of pixels incorrectly labeled as lesions is referred to as true negative (TN). This is classified as a false-positive (FP). False-negative pixels are those that are incorrectly recognized as normal tissue pixels (FN).

## 4. Results Analysis

### 4.1. Performance Comparison of Image Segmentation Model

**Figure 5:**
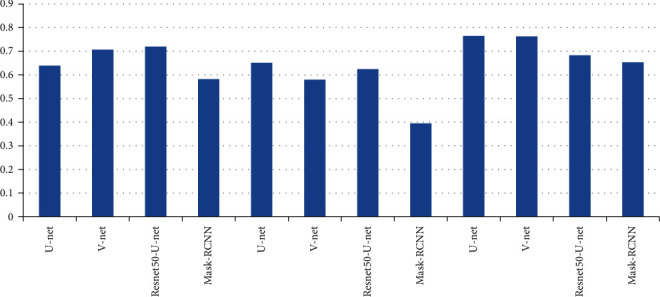
Sensitivity of prostate cancer lesion segmentation.

**Figure 6:**
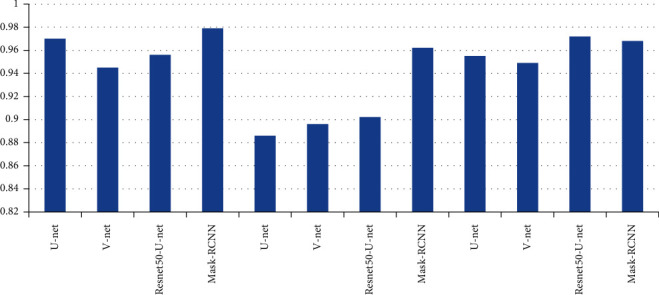
Specificity of prostate cancer lesion segmentation.

Compare the image segmentation network Mask-RCNN [15] based on region classification and three typical image segmentation network models based on pixel classification (U network model [14], V network model [24], and T2W proposed by Literature [25]). Among them, MRCNN uses Resnet50 as a features extraction network, Table 1 and Figure 5, Figure 6 show the segmentation results of different models, where ADC + *T*2W indicates that the corresponding network model concatenates ADC and T2W images into dual-channel images as input for training and testing. As shown in Table 1, when only ADC is used or T2W pictures and when using both ADC and T2W images as model input, MRCNN outperforms pixel-based image segmentation models on DSC, specificity, and PPV. On the other hand, U net, V network model, and Resnet 50-U net Pixel-based image segmentation models such as these outperform MRCNN only insensitivity.

**Figure 7:**
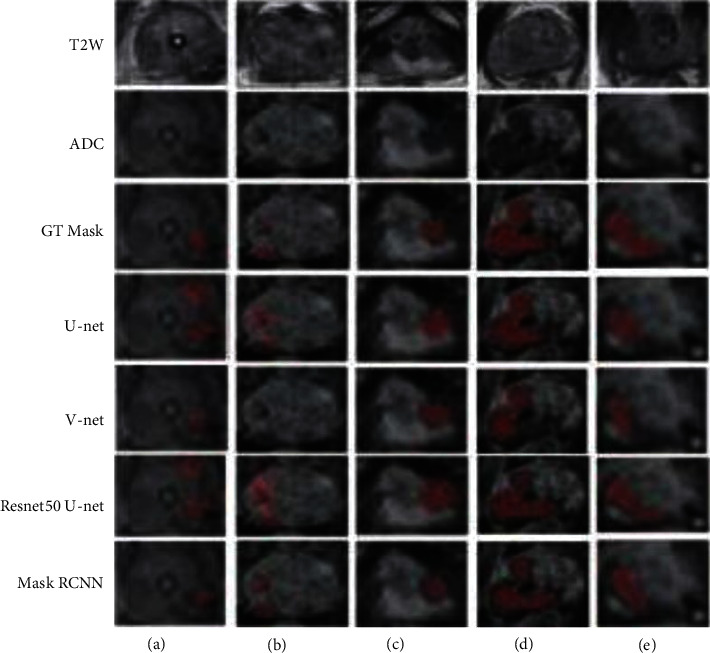
Examples of segmentation results of different network models for mp-MRI prostate cancer lesions.

As shown in Figure 7, to visually display the segmentation results of different network models, the lesion segmentation results of 5 groups of patients were randomly selected. In the figure, the first and second columns are T2W and ADC images, respectively, and the third column is the outline of the doctor. The standard (ground truth, GT) segmentation results of the 4th to 7th columns are U net, V net, Resnet50-U-net, and MRCNN segmentation results.

In Figure 6, the marked area represents the segmented prostate cancer lesion area. Comparing the segmentation results of different models on five patients' tumors, it can be seen that U net, Resnet50-U network model, and V network model have poor segmentation accuracy for prostate cancer margins; there is over-segmentation or under-segmentation. The MRCNN algorithm based on region classification has better segmentation accuracy than another model. The image segmentation methods depend on pixel classification in the task of prostate cancer lesion segmentation play an important role in early detection of prostate cancer. In addition, there are more false-positives in the segmentation results of U network model and Resnet50-U network model in Figure 7(a). Although the sensitivity of the lesion area segmented by the MRCNN model is low, it is suitable for image segmentation models based on pixel classification such as U network model and V net. Still, MRCNN can segment the main lesion area, and there are fewer false-positive areas. Combining the data in Table 1 and the visualization example in Figure 4, it can be seen that, the image segmentation algorithm MRCNN based on region classification has better segmentation accuracy than U net, Resnet50-U network model, and other image segmentation methods based on pixel classification in the task of prostate cancer lesion segmentation.

For the same network structure, the segmentation results of the model trained with ADC and T2W images are superior to the segmentation results of the network model trained with single-sequence MRI in multiple indicators. Therefore, it can be seen that compared with single-sequence MRI, the use of MP-MRI images can effectively improve the automatic segmentation accuracy of prostate cancer lesions. This approach fine-segments the lesions by recognizing and pinpointing the potential locations of prostate cancer lesions. The results of the experiments demonstrate that this strategy can significantly increase the accuracy of automated segmentation of prostate cancer lesions.

### 4.2. SE-Block Improved MP-MRI Information Fusion Method

Aiming at the fact that MRCNN was initially applied to the object detection and segmentation task of natural images, the effective fusion of MP-MRI information was not considered, and an improved mp-MRI information fusion method based on SE-block applied to MRCNN was proposed. To verify the proposed MP-MRI information fusion method, the effectiveness of the MRI information fusion method is compared with the other three MP-MRI information fusion methods commonly used in other studies.

As shown in Table 2, when using Resnet50 as the convolutional network, the segmentation results using the SE-feature map concatenation method are superior to the segmentation results of the other three mp-MRI information fusion methods in terms of DSC, sensitivity, specificity, and PPV. When SE-Resnet was used as the convolutional network, the segmentation results obtained by the SE-feature map concatenation method were the same as those obtained by the input image concatenation method in terms of specificity and PPV; the DSC and sensitivity were significantly higher. Although the segmentation results obtained by using the SE-feature map concatenation method are similar to those obtained by adopting features in terms of sensitivity and PPV indicators, segmentation results obtained by the graph concatenation method are identical. Still, the DSC and PPV are significantly improved. Comparing the feature graph addition method, it can be seen that the segmentation results of the SE-feature graph concatenation method are better in all four evaluation indicators. In the automatic segmentation task of MRI prostate cancer lesions, the segmentation results obtained by the MP-MRI information fusion method using SE-feature map concatenation are better than the segmentation results of the other three mp-MRI information fusion methods. When using SE-Resnet as the convolution, the SE-feature map tandem fusion method achieved the best segmentation results of prostate cancer lesions when using the network.

**Figure 8:**
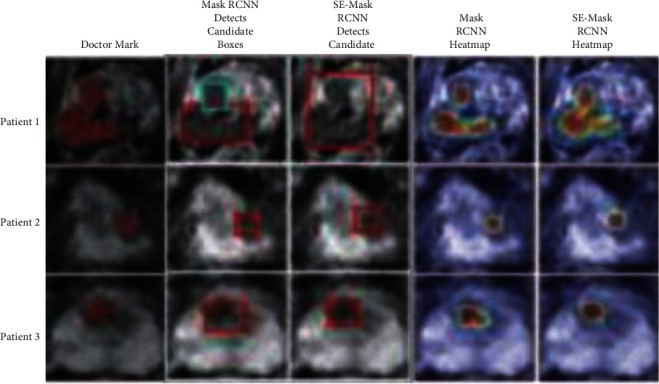
Comparisons of candidate regions and heatmaps.

As shown in Figure 8, to compare the performance of the proposed SEMRCNN and MRCNN in the task of automatic segmentation of prostate cancer lesions, three groups of patient segmentation results were randomly selected as examples. For each patient example, the doctor labels are given, MRCNN and SEMRCNN detected candidate regions and the corresponding heat map. As shown in Figure 8, for patient 1, SEMRCNN can effectively find the candidate regions of prostate cancer lesions. Mask-RCNN divides the lesion into two candidate regions for processing separately, resulting in inaccurate segmentation of the lesion region between the two areas. For patient 2, the candidate region obtained by SEMRCNN can be better than that obtained by Mask-RCNN. Including the entire lesion area, the segmentation result of the lesion edge is better. For patient 3, the candidate area obtained by MRCNN is too large, and the segmented lesion area significantly deviates from the doctor's mark.

SE-Mask-RCNN: The lesion area can be found more accurately, and the segmentation results are closer to the doctor's standard. Combined with the statistical results and intuitive comparison, it can be seen that the proposed SEMRCNN can effectively improve the segmentation accuracy of prostate cancer lesions compared with the original Mask-RCNN.

To explore the reason why the SE-feature map series fusion method is better than using Resnet50 as the convolutional network segmentation result when using SE Resnet as the convolutional network, compare the segmentation results when SE-block in SE-Resnet is removed. As shown in Table 3, ADC SE-Resnet is the volume from which the ADC feature map is extracted in Figure 7(d).

Product network, T2W SE-Resnet is the convolutional network that extracts the T2W feature map in Figure 7(d). Removing SE-block means removing all SE-blocks in the SE-Resnet. It can be seen from Table 3 that when two or when one of the parallel SE-RESNETs removes the SE-block, in addition to a slight increase in sensitivity, the other three indicators are significantly reduced. When the SE-blocks in the two parallel SE-RESNETs are removed, all hands decreased significantly. Many indicators were lower than the segmentation results using Resnet50 as the convolutional network in Table 2.

### 4.3. Model Calculation Time Comparison

**Figure 9:**
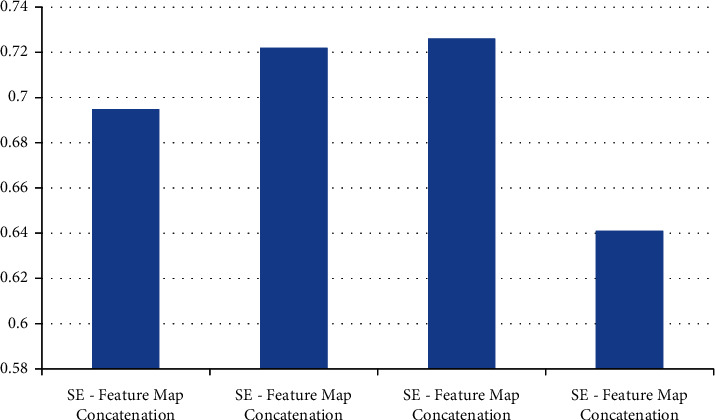
Sensitivity of convolution network SE-block removal methods.

**Figure 10:**
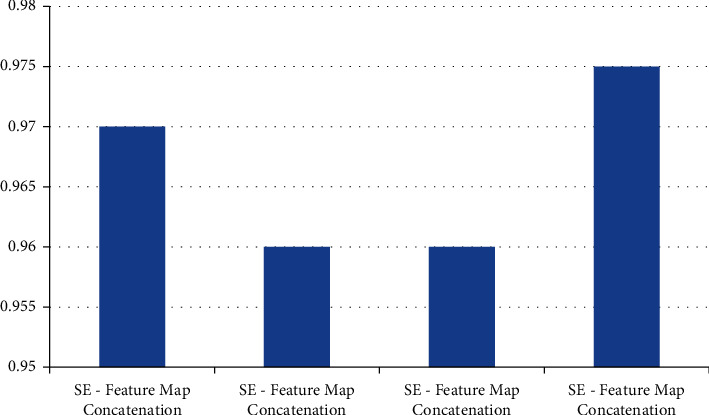
Specificity of convolution network SE-block removal methods.

In the patient data of this study, the number of MRI images in a single sequence of a patient is about 30. This study and other methods automatically segment the prostate cancer lesion time from the prostate region of a single MRI image, as shown in Table 4. Figures 9 and 10 show the sensitivity and specificity for convolution network SE-block removal methods, respectively.

It can be seen from Table 4 that although SEMRCNN has a longer processing time than the other four methods, it only takes 3.0 F02D 4.0 s to process all images of a patient, which is within the acceptable range for clinical use. Therefore, SEMRCNN compared with Mask-RCNN increases the computing time to a small extent and has less impact on clinical use.

## 5. Conclusion

This study proposes an improved deep convolutional network segmentation model SE Mask-RCNN, which can automatically and accurately target prostate cancer lesions on the MP-MRI prostate region. The scope of this research is that the utilization of MP-MRI pictures can effectively increase the automated segmentation accuracy of prostate cancer lesions when compared to single-sequence MRI. This method performs fine segmentation of the lesions based on detecting and locating the candidate regions of prostate cancer lesions, avoiding the interference of other areas, and enhancing the learning of the characteristics of the lesions. Correlation modeling between MRI features is used to enhance useful features and suppress irrelevant features. The location and direction of an image are not encoded by convolutional neural network. It is necessary to collect a large amount of training data. The experimental results show that this method can effectively improve the accuracy of automatic segmentation of prostate cancer lesions.

## Figures and Tables

**Table 1:** Quantitative comparison of the results of different network models for prostate cancer lesion segmentation.

Network model	MRI type	DSC	Sensitivity	Specificity	PPV
U Network model	ADC	0.621	0.685	0.987	0.456
V Network model		0.678	0.859	0.962	0.562
RUNM		0.654	0.802	0.945	0.552
MRCNN		0.652	0.625	0.985	0.621

U Network model	T2w	0.421	0.785	0.845	0.265
V Network model		0.386	0.526	0.823	0.285
RUNM		0.365	0.662	0.956	0.296
MRCNN		0.456	0.456	0.945	0.562

U Network model	Hybrid	0.538	0.856	0.965	0.548
V Network model		0.569	0.852	0.985	0.456
RUNM		0.621	0.785	0.9642	0.526
MRCNN		0.785	0.741	0.947	0.685

**Table 2:** Comparison of four MP-MRI information fusion methods.

Fusion method	Convolutional network	DSC	Sensitivity	Specificity	PPV
Input image concatenation (Mask-RCNN)	Resnet50	0.756	0.756	0.846	0.741
Feature map addition	Resnet50	0.751	0.741	0.845	0.756
Feature map concatenation	Resnet50	0.745	0.754	0.842	0.746
Se - feature map concatenation	Resnet50	0.714	0.753	0.823	0.716
Input image concatenation	Se-Resnet	0.723	0.785	0.863	0.723
Feature map addition	Se-Resnet	0.756	0.795	0.845	0.781
Feature map concatenation	Se-Resnet	0.785	0.712	0.854	0.763
SE-feature map concatenation (SE-Mask-RCNN)	Se-Resnet	0.756	0.715	0.856	0.752

**Table 3:** Performance comparison of Convolutional Network SE-block removal methods.

Fusion method	Whether Adc Se-Resnet removes SE-block	Does T2w Se-Resnet remove SE-block	DSC	Sensitivity	Specificity	PPV
SE-feature map concatenation	No	No	0.654	0.695	0.970	0.685
SE-feature map concatenation	No	Yes	0.633	0.722	0.960	0.634
SE-feature map concatenation	Yes	No	0.633	0.726	0.960	0.630
SE-feature map concatenation	Yes	Yes	0.621	0.641	0.975	0.670

**Table 4:** Average image processing time of different models.

Model	Operation time (ms)
UNM	85
VNM	89
RUNM	86
MRCNN	135
SEMRCNN	156

## Data Availability

The data shall be made available on request.
